# Supplementary material for: Effects of two substrates at different phosphorus levels on morphology and physiology of Dianthus barbatus Linn
Source: PLoS One. 2024 Jun 14;19(6):e0297215. doi: 10.1371/journal.pone.0297215 (PMC11178218; doi:10.1371/journal.pone.0297215)
Supplement: Attachment — Submitted filename: Response to Reviewers.docx [file pone.0297215.s003.docx]

December 6, 2023

Dr. Mojtaba Kordrostami, Ph.D. Editor, PLOS ONE

**Re:** Response for manuscript “Effects of two substrates at different phosphorus levels on morphology and physiology of Dianthus barbatus Linn”

Dear Dr. Mojtaba Kordrostami, Ph.D. Editor, PLOS ONE,

Thanks for providing us with this great opportunity to submit a revised version of our manuscript. We appreciate the detailed and constructive comments provided by the reviewers. We have carefully revised the manuscript by incorporating all the suggestions by the review panel.

We hope this revised manuscript has addressed your concerns, and look forward to hearing from you.

Sincerely,

The Authors

Encl. Responses to the comments from Reviewer 1, 2.

**Reply to Reviewer #1**

Dear Reviewers,

Thank you very much for your time involved in reviewing the manuscript and your very encouraging comments on the merits. We also appreciate your clear and detailed feedback and hope that the explanation has fully addressed all of your concerns. In the remainder of this letter, we discuss each of your comments individually along with our corresponding responses.

To facilitate this discussion, we first retype your comments in *italic font* and then present our responses to the comments.

**Comments 1:**

*“In this manuscript, what is the normal level of phosphorus supply for Dianthus?”*

**Response 1:**

Thank you for your detailed review. In the "Introduction", we emphasize finding the most suitable phosphorus concentration for *D. barbatus* growth and improving the absorption and utilization efficiency of phosphorus by *D. barbatus*. In "Materials and Methods", we mentioned that the blank treatment of this experiment used untreated soil. The loess soil has the following components: soil total phosphorus was 0.47 g/kg. The vegetable soil has the following components: soil total phosphorus was 0.85 g/kg.

**Comment 2:**

*In the experiment, are the different phosphorus concentrations used considered high or low levels for Dianthus?*

**Response 2:**

Thanks for your great suggestion on improving the accessibility of our manuscript. We are very sorry for the trouble caused to you. Under low-(0.192${g\cdot Kg}^{-1}$) and medium-to-low-P(0.384${g\cdot Kg}^{-1}$) treatments treatments, the indicators of both aboveground and underground parts improved and promoted plant growth, and higher or lower than this range will not be conducive to the growth of *D. barbatus*.

**Comment 3:**

*Please use one paragraph to introduce the main methods, kits, and calculation formulas in the "Soil acid phase activity" section. Do not describe trivial content such as" preparation" and "procedure", and etc.*

**Response 3:**

Thanks for your great suggestion on improving the accessibility of our manuscript. We are very sorry for the trouble caused to you. We have deleted the words "preparation" and "procedure" in the section "Soil acid phase Activity". And retain the parts that make the essay coherent.

**Comment 4:**

*What dose Fig.9 mean?*

**Response 4:**

Thanks for your great suggestion on improving the accessibility of our manuscript. We are very sorry for the trouble caused to you. The purpose of Figure 9 is to enhance readers' comprehension of *D. barbatus* by presenting photographs depicting their growth under varying phosphorus conditions. These images were captured in October 2021 and April 2022. The darker color is vegetable soil, and the other is loess.

**Comment 5:**

*Additionally, the manuscript should also pay attention to writing standards.*

**Response 5:**

Thanks for your great suggestion on improving the accessibility of our manuscript. We have modified the article according to the style and formatting guidelines.

We would like to take this opportunity to thank you for all your time involved and this great opportunity for us to improve the manuscript. We hope you will find this revised version satisfactory.

Sincerely,

The Authors

**Reply to Reviewer #2**

Dear Reviewers,

Thank you very much for your time involved in reviewing the manuscript and your very encouraging comments on the merits and this great opportunity for us to improve the manuscript. We hope you will find this revised version satisfactory.

Sincerely,

The Authors
